# Food and Beverage Advertising to Children and Adolescents on Television: A Baseline Study

**DOI:** 10.3390/ijerph17061999

**Published:** 2020-03-18

**Authors:** Adena Pinto, Elise Pauzé, Rachel Mutata, Marie-Hélène Roy-Gagnon, Monique Potvin Kent

**Affiliations:** School of Epidemiology and Public Health, University of Ottawa, Ottawa, ON K1G 5Z3, Canada

**Keywords:** child obesity, food and beverage marketing, television advertising, food policy, nutrition policy

## Abstract

The progressive rise in Canadian child obesity has paralleled trends in unhealthy food consumption. Industry has contributed to these trends through aggressive food and beverage marketing in various media and child settings. This study aimed to assess the extent of food and beverage advertising on television in Canada and compare the frequency of food advertising broadcasted during programs targeted to preschoolers, children, adolescents and adults. Annual advertising from 2018 was drawn from publicly available television program logs. Food and beverage advertisement rates and frequencies were compared by, target age group, television station, month and food category, using linear regression modelling and chi-square tests, in SAS version 9.4. Rates of food and beverage advertising differed significantly between the four target age groups, and varied significantly by television station and time of the year, in 2018. The proportion of advertisements for food and beverage products was significantly greater during preschooler-, child-, and adult-programming [5432 (54%), 142,451 (74%) and 2,886,628 (48%), respectively; *p* < 0.0001] compared to adolescent-programming [27,268 (42%)]. The proportion of advertisements promoting fast food was significantly greater among adolescent-programming [33,475 (51%), *p* < 0.0001] compared to other age groups. Legislation restricting food and beverage advertising is needed in Canada as current self-regulatory practices are failing to protect young people from unhealthy food advertising and its potential negative health effects.

## 1. Introduction

Canadian child obesity has increased at an alarming rate, nearly tripling in the last three decades [1]. As of 2012, 30% of Canadian children, aged 5–17, had excess weight or obesity [2]. The progressive rise in childhood obesity, however, is not exclusive to Canada, and worldwide an estimated 108 million children (under 20 years old) suffer from obesity [3,4]. Health conditions, previously only common in adults (e.g., Type 2 diabetes and cardiovascular disease), have also become increasingly prevalent among children with obesity [5]. One major driver, increasing child and adolescent consumption and preference for foods high in sugar, sodium and saturated fats, is the food industry’s aggressive food and beverage marketing in various media and settings [6]. The World Health Organization (WHO) defines marketing as “any form of commercial communication or message that is designed to, or has the effect of, increasing the recognition, appeal, and/or consumption of particular products and services” and also includes “anything that acts to advertise or otherwise promote a product or service” [7]. Food and beverage marketing, thereby, refers to the activities designed to promote food and beverage products and services [8].

Television continues to be a major source of food and beverage marketing to children and adolescents, as Canadian youth watch between 14 to 17 h of television per week [9]. Research shows television-based food and beverage marketing directed at children predominantly promotes unhealthy foods (e.g., sugary breakfast cereals, fast-foods, candy, etc.) [10,11]. A recent study, conducted by Kelly et al., revealed that among 22 countries, Canada had the highest rate of food and beverage advertising during children’s viewing times. Approximately 11 food and beverage advertisements aired per hour, per channel; the majority of which promoted breakfast cereals, candy, and cakes, cookies and pastries [12]. Canadian studies have also shown that fast food is heavily advertised during programs viewed by children and adolescents [13,14,15]. This exposure to unhealthy food and beverage advertising has previously been associated with a higher intake of energy-dense, sweet and salty foods among those exposed [16,17,18,19,20]. Experimental evidence reveals child-oriented food and beverage television advertising also influences children’s food requests and has been associated with increased pestering of parents to purchase advertised products, otherwise known as “pester power” [20,21,22]. Adolescents and young adults, however, are autonomous in their spending habits and are a particularly valuable target for fast food marketing, due to their spending power [23].

The WHO has responded to concerns about the impact of food and beverage marketing practices on child and adolescent health, by urging countries to regulate and limit the marketing of foods and non-alcoholic beverages high in fat, sugar, and salt, directed at children [19,24,25]. In Canada, food and beverage marketing is largely self-regulated by Ad Standards Canada and the food industry [26,27]. All Canadian television broadcasters must adhere to the Broadcast Code for Advertising to Children, and its Code Interpretation Guidelines, which state that advertised foods must be within the context of a balanced meal, depict a reasonable serving size for a child, and must not portray snack foods as substitutions for meals nor discourage foods recommended by Canada’s Food Guide to Health Eating (e.g., fruits or vegetables) [27,28,29]. The Code and the Children’s Broadcast Advertising Clearance Guide also clearly indicate advertising to preschoolers is not permitted in Canada [27,28]. In 2007, the Canadian Children’s Food and Beverage Advertising Initiative (CAI) was launched, and currently comprises of 16 large Canadian food and beverage corporations who have voluntarily pledged to either refrain from advertising unhealthy food and beverages to children under 12 years old entirely, or advertise only what the companies define as “better-for-you” products. The latter must comply with the CAI’s Uniform Nutrition Criteria (implemented in 2015), on television and in other media [6,30,31,32,33]. Despite the CAI’s efforts to limit food and beverage advertising directed at children, under 12 years old, studies have shown this self-regulatory program has insufficiently protected Canadian children from unhealthy food and beverage advertising on television [13,34,35]. Critiques of the CAI report that CAI companies are responsible for more food and beverage advertising during children’s viewing than non-CAI participating companies, and that up to 58% of its “better-for-you” advertised products (e.g., Froot Loops) fail to pass the nutrition standards of many national governments [14,36]. Other criticisms levied at the CAI include child-audience thresholds (i.e., the percentage of the viewing audience that must be children, under 12 years old, before the pledges are implemented) that are too high, low participation rates; and narrow definitions of child-directed marketing [35].

The ineffectiveness of the CAI in Canada led to the development of more comprehensive and stringent legislation—Bill S-228, The Child Health Protection Act. This Bill, introduced in 2016, sought to amend the Food and Drugs Act by prohibiting unhealthy food and beverage marketing directed primarily at children under 13 years old, across all potential marketing media channels and child settings in Canada [37]. Bill S-228, however, died on the order paper during the dissolution of Parliament, prior to the 2019 Canadian federal election. Given that this Bill (or something similar) may be reintroduced in the current Parliamentary session, and given the deleterious effects of unhealthy food advertising on child and adolescent health, it is necessary to benchmark the current food and beverage advertising environment for policy makers. The current literature, however, is limited with respect to Canadian television food advertising directed at preschoolers and adolescents, across the 12-month calendar year, and on television stations intended for general audiences [6,12,13]. To assess the impact and effectiveness of new food and beverage marketing legislation, national and yearlong baseline data on the current state of television food advertising to children and other youth under 18, is needed.

The purpose of the current study was to benchmark the frequency of food and beverage advertising on television over a one year period and to examine differences in advertising, overall and by food category, between television programming targeted to preschoolers (0–5 years old), children (6–12 years old), adolescents (13–17 years old) and adults (18 years and older). It was predicted that programs targeted to adolescents would have the highest rates of food advertising compared to other programs, while programs intended for preschoolers would have the lowest rates of food advertisements. It was also hypothesized that programs targeted to adolescents would have greater proportions of fast-food restaurant advertisements than programs targeted to other age groups.

## 2. Materials and Methods

This baseline study performed an analysis on the Canadian Radio-television and Telecommunications Commission’s (CRTC) television program logs, which are submitted by television broadcasters, and collected and published by the CRTC on the Government of Canada’s open data portal [38]. These logs are open-access monthly television programming records which report elements of programming and advertising content including, but not limited to, the broadcast date, duration, program titles, and target age group of programs, for over 300 Canadian television stations, in compliance with the Television Broadcasting Regulations, 1987. Importantly, broadcasters are required to report the names of all individuals or companies promoting goods and services on Canadian television stations [39]. The study examined data on television programming and food advertising airing between 6 a.m. and midnight (when most young people under 18 are watching television) from January to December 2018 on 271 commercial stations. Of the television stations reported in 2018, one station (BBC Kids) that was discontinued in 2019, one station (VisionTV) that had missing data on target age groups and 39 television stations that did not air advertisements were excluded.

### 2.1. Measures

The unit of observation was “advertising spots” which the Television Broadcasting Regulations, refers to as programming content consisting of commercial messages and programing used for the promotion of stations, networks, or programs [39]. For this study, advertising comprised of commercial messages, giveaways, local advertising, merchandising, solicitation messages, and sponsorship messages, identified from the CRTC program logs’ “Programming Class ID” variable. Commercial message was defined as “an advertisement intended to sell or promote goods, services, natural resources or activities … that is broadcast in a break within a program or between programs” [40]; local advertising referred to less than 12 min of advertising that is “of interest to the community or market served” [41]; giveaway referred to advertising where products are given free, for promotional purposes [42]; merchandising consisted of advertising material promoting the sale of goods [43,44]; solicitation messages was advertising involving “the sale or promotion of a product or service … on behalf of another group” [45]; and finally, sponsorship messages consisted of advertising “when a community program acknowledges that it has received direct financial assistance” [46].

Within the program logs, pertinent variables associated with advertising spots included “log date,” which was the recorded date when advertisements and programs were aired; “start time” and “end time,” which referred to the recorded time of the day (in terms of hours, minutes, and seconds) when advertisements and programs were aired; “duration” consisted of the recorded length of the advertisement and programs (in terms of hours, minutes, and seconds); “call sign” or “log identifier,” which identified television stations; and finally, the “program title,” which indicated the name of the broadcasted program or advertiser and the advertised product or service [39].

According to the CRTC, commercial messages airing on children’s programming are considered to be targeted to children (defined as children under 12 years of age) [29]. The “target age group” of advertisements aired during and immediately adjacent to television programs was, thus, determined from the target age group of television programs. Note that the target age group of every television program is selected by each broadcaster (i.e., the television station). They must classify each television program as targeting one of four age groups defined by the CRTC as preschoolers (0–5 years old), children (6−12 years old), adolescents (13–17 years old) and adults (18 years and older) [47].

Data from the program title (i.e., advertised product or company) was used to identify advertisements as food or non-food, and the food category. Food and beverage advertising included food-related promotions which comprised of the following food categories: food and beverage products (excluding gum and alcohol), diet services and related products (e.g., Weight Watchers), food delivery services and applications (e.g., Skip the Dishes), meal kits and subscription services (e.g., Hello Fresh), restaurants (e.g., sit-down restaurants, diners, cafes, etc.), fast food restaurants (i.e., quick-service restaurants where food is ordered from a menu board, purchased at a counter and taken to a table or elsewhere by the customer), retail-outlets (e.g., grocery stores, bakeries, farmer’s markets, etc.), and unknown. In cases where the advertised product was not specified, and companies manufactured both food and non-food products (e.g., Unilever Canada, Mars Inc. etc.), ads were classified as non-food and omitted from the analysis.

### 2.2. Data Handling

Television program logs, from January to December 2018, were downloaded from the Government of Canada’s Open data portal on July 4, 2019 and imported into SAS version 9.4 for Windows (SAS Institute, Released 2013) [38,48]. As the formatting of the program log data changed in 2018, the television station, program class and target age group variables were converted to the numeric data format, using the Reference Tables provided by the CRTC, to append the two formats of data into a full year dataset [49]. A total of 293 numeric and character station codes were then classified into 271 stations (see Supplementary Table S1 for all included stations), consisting of both child-specialty stations (i.e., stations whose content is specifically intended/targeted at children under 18 years old) and non-child-specialty stations (i.e., stations whose content is not specifically intended/targeted at children under 18 years old) [50].

To identify food and non-food related advertisements, a code was created using keywords from the advertisement titles and applied to the full year dataset. Advertising titles were examined individually to prevent and correct misclassification of advertisements. Notably, any non-food ads that remained in the dataset, after extracting food ads, were deleted to obtain a full-year dataset consisting of only food and beverage advertisements and television programs. Food categories (mentioned above) were identified for each food advertisement using the same methodology, within the dataset containing only food advertisements and television programs. For example, from the advertisement title “GENERAL MILLS CANADA CORP” we identified the keyword “General Mills” and classified the advertisement as a food ad in the “food and beverage products” category.

### 2.3. Statistical Analysis

Descriptive statistics and regression modelling examined the association between target age group and rate of food and beverage advertising, on SAS version 9.4 (SAS Institute Inc., Cary, NC, USA) for Windows [48]. The rate of food and beverage advertising, defined as the average number of food and beverage-related advertisements aired per hour of programming (n/hour), was calculated for each target age group at the month and television station level. The frequency of food and beverage television advertising by food category, defined as the number and proportion of advertisements from a specific food category, was also calculated and examined separately for each target age group. The effect of target age group on the average rate of food and beverage advertising was tested using simple and multiple linear regression models. A simple linear regression model observed the unadjusted effect of target age group on food advertising rates and tested differences in the annual age-specific rates of food advertising. Multiple linear regression models were fitted to determine the influence of target age group, television station, and month on food advertising rates and to test differences in the station- and month-specific food advertising rates between the four target age groups (using an ESTIMATE statement). A full regression model, including all 271 television stations, was fit to examine monthly differences in the rates of food advertising between the four target age groups. Age-specific monthly rates were also plotted to observe seasonal variations in advertising over the course of 2018. A specific regression model, containing 31 television stations (14 child-specialty stations and 17 of the top five non-child-specialty stations), was fit to examine significant differences in the station-specific rates of food advertising between the four target age groups. Notably, the top five non-child-specialty stations were determined by selecting five television stations with the highest food advertising rates for each target age group. The specific model excluded Disney XD, Citytv Montreal and Zeste, as each of these stations carried programming for only one target age group. Pearson’s chi square analysis was used to test differences in the proportion of food and beverage advertisements of each of the food categories among the four target age groups. Statistical significance level was set at α < 0.05, and significant results are reported using a 95% confidence interval and/or *p*-values less than 0.05. A sensitivity analysis was conducted, by inputting advertisements from companies manufacturing both food and non-food products, which either lacked or had unclear advertised product information, into the chi square analysis and regression models, to determine whether our method and assumptions for handling unclear advertising data influenced the magnitude and direction of our estimates.

## 3. Results

### 3.1. Annual Results

In 2018, 6,344,557 food and beverage-related advertisements and 3,575,558 programs (i.e., TV series, films, etc.) were broadcasted on 271 Canadian television stations. As shown in Table 1, 96% of food advertising was broadcasted during programs targeted to adults (18 years and older), 3.1% was broadcast during programs targeted to children (6−12 years old), 1.0% was broadcast during programs targeted to adolescents (13−17 years old) and 0.2% was broadcast during programs targeted to preschoolers (0−5 years old). Across all stations, the average annual rates of food advertising differed between the age groups at 0.6 food ads per hour of programming targeted to preschoolers; 1.5 food ads per hour of programming targeted to children; 3.3 food ads per hour of programming targeted to adolescents; and 4.1 food ads per hour of programming targeted to adults.

A simple linear regression model estimated the unadjusted effect of the target age group on the average annual food advertising rate (Table 1). A significant regression equation was found with only 13.9% of the variation (R^2^ = 0.139, *p* < 0.0001) explained by the target age group alone. In 2018, the average annual rate of food advertising was significantly lower when compared to programs targeted to adults, by 3.5 food ads per hour when programs were targeted to preschoolers (β = −3.5, *p* < 0.0001); by 2.6 food ads per hour when programs were targeted to children (β = −2.6, *p* < 0.0001); and by 0.7 food ads per hour when programs were targeted to adolescents (β = −0.7, *p* < 0.0001).

### 3.2. Monthly Advertising Rates

**Figure 1:**
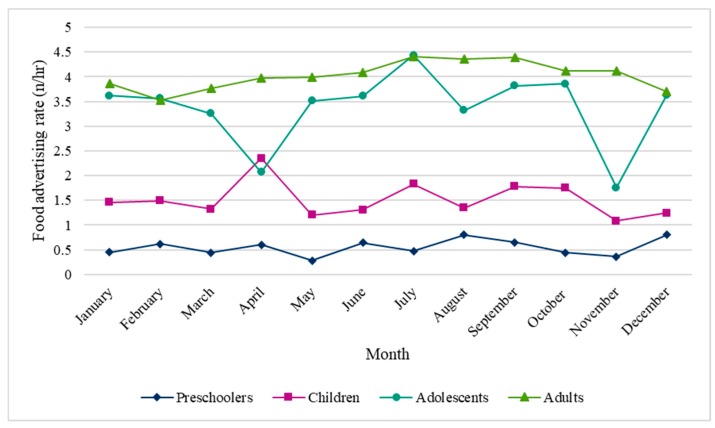
Monthly age-specific food advertising rates, in terms of food ads per hour of programming, on Canadian television stations, in 2018.

The average annual rates of food advertising, however, may mask seasonal variations found in food advertising rates across the year. As depicted in Figure 1, food advertising rates during programs targeted to preschoolers, children and adults were relatively consistent throughout the year. In April, however, a large peak was found in the food advertising rate during programs targeted to children, likely due to the station W Network, which aired 36 food ads (approximately 10 min) during and immediately following a single 28-min program targeted to children. This translated to a food advertising rate of 77 food ads per hour of programming (approximately 23 min of food advertising/hour) targeted to children. Conversely, large fluctuations in rates of food advertising were observed during programs targeted at adolescents, across the 12-month period. Among programs targeted to adolescents, the highest food advertising rate was observed in July (approximately 4 food ads/hr), likely due to the station YTV, which had 21 food ads per hour of programming (approximately 9 min of food advertising/hour). Other peaks in food advertising during programs targeted to adolescents coincided with months with major commercialized holidays, including February, October and December (i.e., Valentine’s, Halloween, and Christmas, respectively).

A full multiple linear regression model determined the influence of target age group, time of the year (i.e., month) and television station (all 271 stations) on food advertising rate. A significant regression equation was found with 91.0% (R^2^ = 0.910, *p* < 0.0001) of the variation explained by target age group, month and television station. Significant interactions were found between target age group and television station, and between target age group and month, indicating the effect of the target age group on food advertising rates was modified by the television station and time of the year (i.e., month). Notably, the month of May was used as the reference due to its absence of, and distance from, major food-related holidays. Significant differences in food advertising rates between May and other months were only found for adolescent-targeted programs. Among programs targeted to adolescents, compared to the month of May, the rate of food advertising was significantly lower in April and November by an average of 1.30 food ads per hour (95% CI = −1.76, −0.84) and 0.78 food ads per hour (95% CI = −1.26, −0.31), respectively, while television stations remain constant. Significant differences in monthly rates were not observed between other months or target age groups.

### 3.3. Station-Specific Advertising Rates

Rates of food advertising also varied substantially by television station (all station-specific rates are found in Supplementary Table S1). On child-specialty stations, the rates of food advertising were higher among programs targeted to adults compared to programs targeted to younger age groups, with the exception of ABC Spark, Nickelodeon, Teletoon (English), VRAK and YTV, which had higher rates of food advertising during programs targeted to children or adolescents (Table 2). Notably, only YTV had statistically significantly higher rates of food advertising during programs targeted to adolescents compared to programs targeted to adults.

Average rates of food advertising, however, were significantly higher during programs targeted to preschoolers, children and adolescents, on many non-child-specialty stations (Table 3). Among the top five non-child-specialty stations for preschoolers, OMNI Toronto had the highest food advertising rate during programs targeted to preschoolers, at 11 food ads per hour. The highest food advertising rate during programs targeted to children and adolescents was found on Citytv Toronto at 20 food ads per hour and on Lifetime at nearly 19 food ads per hour, respectively. Among programs targeted to adults, the highest rate was found on The Cooking Channel at 15 food ads per hour.

A specific multiple linear regression model was fit to estimate the effect of target age group, television stations and months on food advertising rates between the four target age groups, across 31 television stations (specific model found in Supplementary Table S2). A significant regression equation was found with 89.6% of the variation (R^2^ = 0.896, *p* < 0.0001) explained by target program age, television station, and month. Significant interactions were also found, in the specific regression model, between target age group and television station, and between target age group and month. Among child-specialty stations, statistically significant differences were found between food advertising rates during programs targeted to adults and programs targeted to younger people on Cartoon Network, Disney Channel English, Family Channel, Teletoon English and French, Treehouse TV, and YTV. For instance, on Disney Channel English, compared to programs targeted to adults, the rate of food advertising significantly decreased by 3.0 food ads/hour (95% CI = −4.37, −1.63) when programs are targeted to preschoolers, by 1.8 food ads/hr (95% CI = −3.15, −0.41) when programs are targeted to children and by 2.6 food ads/hr (95% CI = −3.99, −1.25) when programs are targeted to adolescents, while month remains constant. On YTV, compared to programs targeted to adults, the rate of food advertising significantly increases by 2.6 food ads/hr (95% CI = 1.09, 4.17) when programs are targeted to adolescents, but decreases by 9.2 food ads/hr (95% CI = −11.7, −6.62) and 5.6 food ads/hr (95% CI = −6.93, −4.32) when programs are targeted to preschoolers and children, respectively, while month remains constant.

Significant differences in food advertising rates between the four age groups were also observed for several non-child-specialty television stations (specific model in Supplementary Table S2). The rate of food advertising significantly increases by 5.4 food ads/hr (95% CI = 3.51, 7.29) on OMNI Toronto and 3.7 food ads/hr (95% CI = 0.22, 7.15) on Sportsnet One and significantly decreases on Global Kelowna by 4.0 food ads/hr (95% CI = −7.40, −0.51) when programs are targeted to preschoolers compared to when programs are targeted to adults, while month remains constant. On Citytv Toronto, Showcase and Yes TV Burlington, the food advertising rate significantly increases by 11.0 food ads/hr (95% CI = 7.60, 14.42), 2.8 food ads/hr (95% CI = 1.21, 4.44), and 4.8 food ads/hr (95% CI = 3.49, 6.10) when programs are targeted to children compared to when programs are programs targeted to adults, while month remains constant. On Lifetime, Citytv Vancouver and Slice, the food advertising rate significantly increases by 7.2 food ads/hr (95% CI = 3.78, 10.71), 7.3 food ads/hr (95% CI = 3.77, 10.71) and 3.4 food ads/hr (95% CI = 1.73, 4.99), respectively, when programs are targeted to adolescents compared to when programs are targeted to adults, while month is held constant.

Television food advertising also differed significantly between program target age groups by food category. Results of the Pearson’s chi square analysis (Table 4) indicate the frequency and proportion of food and beverage advertising by food category, was significantly different (χ^2^ = 66128.1; df = 21; *p* < 0.0001) between program target age groups, in 2018. Among programs targeted to preschoolers and children, the majority of food advertising (53.7% and 73.5%, respectively) were promotions for food and beverages, which included items such as snack foods, candy, and breakfast cereals. Food advertising during programs targeted to adolescents had a significantly greater proportion of fast food restaurant advertisements (51.1%) than any other food category. Food advertising during programs targeted to adults were most frequently food and beverage products (47.5%), followed closely by fast food promotions (39.0%).

### 3.4. Sensitivity Analysis

Results of the sensitivity analysis were consistent with the overall primary analysis. The inclusion of advertisements lacking or providing unclear reporting of advertised products by companies including, but not limited to, Unilever, J.M. Smucker, Mars Inc. and Nestle, did not significantly alter the magnitude, direction of, or differences between food advertising rates and frequencies. In fact, the results of our sensitivity analysis indicate food advertising rates are influenced by program target age group, television station, time of the year (i.e., month), and food category to the same degree as when unclear advertisements were omitted from the analysis.

## 4. Discussion

This study adds to the body of literature examining television food advertising directed at children and adolescents, by benchmarking the current television food advertising environment in Canada. As hypothesized, programs targeting preschoolers had the lowest average rates of food advertising across all television stations examined, while, contrary to what was hypothesized, programs targeting adults had the highest average rates of food advertising across most of the examined Canadian television stations, even child-specialty stations. Nevertheless, programming targeted at youth under 18 years had sizeable annual levels of food and beverage advertising as programs directed at preschoolers, children and adolescents had 0.6 ads/hr, 1.5 ads/hr, and 3.3 ads/hr on average, respectively. The average rates of food advertising for children found in our study are lower when compared to previous Canadian studies, which observed between 3−11 food ads per hour per television station [11,33,51]. These differences, however, could be explained by the use of differing methodologies. In particular, previous studies have examined food advertising rates either during all programming on child specialty stations or during programming where children aged 2−11 constituted a large share of the audience (>35%). Our study, by contrast, analyzed food advertising during programming that was classified as targeted to children, or other age groups, by the broadcaster and no criteria for this categorization is publicly available.

### 4.1. Variability among Stations

Our study found that average rates of food advertising varied significantly by television station, in Canada. Non-child specialty stations had especially high average rates of food advertising during programs targeted to youth under 18 years, such as City TV Toronto and Lifetime, which had an average of 20 food ads per hour of programming targeted to children and 19 food ads per hour of programming targeted to adolescents, respectively. Child-specialty stations, conversely, had significantly lower rates of food advertising during programming directed at youth under 18, with the exception of ABC Spark, Nickelodeon, Teletoon (English), VRAK and YTV. These child-specialty stations, particularly Nickelodeon, Teletoon and YTV, have previously been reported as having high rates of food advertising in Canada [12,13,15,35]. Importantly, as YTV is offered in most expanded basic TV packages across Canada, its high level of food advertising is likely due to the greater access to, and viewership of, this station. It has been reported that YTV and Teletoon are the most preferred television stations among children and adolescents (9–15 years old) across Canada (excluding Québec), while VRAK and Teletoon are particularly popular among children and adolescents (9−18 years old) in Québec [52]. Some child-specialty television stations, however, do confer protection to certain age groups and not others (e.g., Disney Channel French, YOOPA), while some stations protect all ages (e.g., Family Jr, Disney Jr) as they do not advertise food-related products and services at all.

### 4.2. Food Advertising Violations

Despite the low rates of food advertising among preschooler-targeted programing found in our study, the majority of child-specialty stations and several non-child-specialty stations (i.e., general audience stations), advertise during programming classified as targeted at preschoolers, which is a clear violation of the Code’s preschooler advertising regulations. Moreover, the Code explicitly states that a station or network may not carry more than 8 min of commercial advertising per hour of children’s programming [27]. This clause was violated by W Network, who aired almost three times the allowed duration of food advertising during child-targeted programs in April 2018. These results point to a need for systematic surveillance of the Code to ensure company and broadcaster compliance.

### 4.3. Food Advertising to Adolescents

Food advertising during adolescent-targeted programming is also a growing concern in Canada. Between 2006 and 2011, Potvin Kent et al. found a significant increase (67%) in the frequency of advertising to adolescents, by 18 CAI companies alone [13]. Our results found the rates of food advertising during adolescent-targeted programming to be alarmingly similar to rates during adult-targeted programming. This is concerning as adolescents are not immune to the effects of food marketing [53]. In fact, not only are adolescents less likely to be critical of food advertising than adults, but this age group is also highly targeted due to their purchasing power [17,53]. Food companies strategically advertise to adolescents, as evidenced by the elevated rate of food advertising during adolescent-programming in July 2018 (i.e., summer vacation); the time during which they are more likely to be watching television. Food advertising to this age group is also likely to be less healthy compared to other age groups. As hypothesized, the proportion of fast food advertising was highest during adolescent-targeted programming, compared to all other food categories and other target age groups. One plausible explanation for this result could be related to adolescents’ increased autonomy in terms of purchasing fast food [54]. Since fast food is affordable for adolescents, it has been suggested that purchasing fast food becomes “a form of self-expression for teenagers as they struggle to assert their autonomy apart from their family’s food identity” [54]. Adolescents, thus, are a highly profitable demographic for fast food companies. In fact, data on food advertising expenditure to youth, in the United States, has revealed fast food companies spend more on advertising to reach adolescents compared to children [55].

Food and beverage product advertisements, on the contrary, are more common in younger children (aged 12 or younger) due to “pester power.” As younger children are unlikely to possess the ability to purchase food outside the home, they contribute to household food purchases through parental requests, which can be exploited by food and beverage manufacturers. Our study demonstrates Canada’s industry-led food advertising initiative (i.e., the CAI) is failing, evident from the persistent high rates of food advertising to children under 12, despite its introduction over a decade ago. Previous research has also shown that CAI companies continue to advertise to children under 12, despite their pledges to abstain from advertising when children make up 25−35% of the viewing audience [35]. Moreover, the CAI fails to capture the breadth of food companies advertising to children under 12 years old. Of the 16 food companies currently participating in the CAI, only one fast food company (i.e., McDonald’s) participates.

### 4.4. Fast Food Advertising

The abundance of fast food advertisements broadcast during programming targeted to youth under 18, observed in our study, is alarming and mirrors what has been repeatedly seen in other Canadian studies on food marketing [13,14,15]. Most recently, a study found a 25% increase in fast food advertising between 2011 and 2016 across 31 stations broadcasted in Toronto, Canada’s largest broadcast market [56]. Fast food advertisements have previously been associated with an increase in fast food consumption, and an increased risk of obesity in children [21,57,58,59,60]. Specifically, studies have found children preferred foods when they thought foods were from familiar fast food brands (e.g., McDonald’s) [61,62]. Other experimental research has shown that fast food advertisements promoting healthy meals increased children’s general preference for fast food, but not for healthier options [63]. As regular consumption of fast food has also been found to increase one’s risk of metabolic syndrome, cardiovascular disease, and developmental diabetes, the volume of fast food advertising during programming targeted at youth under 18, in Canada, is cause for concern [64].

### 4.5. Strengths & Limitations

The main strength of this study is that it is a broad assessment of the Canadian food advertising landscape using the most comprehensive public advertising data available in Canada. This study is the first to investigate and benchmark food advertising rates on nearly all Canadian commercial television stations over a one-year period; assess food and beverage advertising during programming targeted to preschool children and adults, an area of research relatively non-existent; analyze seasonal variations in food advertising; and finally, one of the first to use publicly available data (i.e., CRTC TV program logs) to examine children’s potential exposure to food advertising. Furthermore, this study is one of the first to examine food advertising rates using regression modelling, thereby, enabling us to investigate a greater number of factors that impact the effects of target age on food advertising rates. Our sensitivity analysis also increases the confidence in our data handling assumptions and the robustness of our study findings.

Despite these strengths, we have likely underestimated the rates of food advertising on television in Canada, as the CRTC program logs do not consistently report the food advertising company and/or advertised food product or service clearly. Moreover, rates of food advertising during programs targeted to younger age groups are likely underestimated due to the fact that broadcasters are required to select only one of the four target age groups for each program, while food advertisements carried during these programs may cut across more than one target age group. It is also known that younger children, particularly those of preschool years, actively and passively watch programs intended for other age groups. As such, although stations indicate the program and corresponding food advertisements as targeted to one age group, this does not mean that only the reported target age group views, or is influenced by, the food advertisements.

The reproducibility of our study’s results is also impacted by the availability and changes of 2018′s TV program logs. During our analysis, we observed differences in the number of station files downloaded on different dates (hence the reported date of download in the methods section above). We also found that average rates of food advertising differ depending on the calculation. For example, the average food advertising rate during child-targeted programming calculated from the annual total of child-targeted food advertisements and total child-targeted programs ($\mu =\frac{\Sigma \;child-targeted\;food\;ads\;in\;2018}{\Sigma \;\mathrm{child}-\mathrm{targeted}\;programs\;in\;2018\;}\;$), was different from the mean rate of food advertising during child-targeted programming which was calculated by averaging the food advertising rates across all stations and months ($\mu =\frac{\Sigma \;child-targeted\;advertising\;rates\;across\;all\;stations\;and\;months}{number\;of\;child-targeted\;food\;advertising\;rates\;in\;2018}$). The latter is preferred as it incorporates variance from television stations and months and, thereby, provides more insight into the variations in average food advertising rates. Finally, as there are no published criteria on how broadcasters classify the target age group of their programming, the validity of this classification is unknown.

We are unable to determine actual exposure to food advertising on television, from our analysis, as young people may be watching programming ostensibly only intended for older age groups or using other personalized viewing services offered by television service providers (e.g., pay-per-view and/or video on demand). Nevertheless, this research provides insights on children’s potential exposure to food and beverage marketing on television as well as Canadian broadcasting practices and their protection of young people from food and beverage advertising, or lack thereof. While we were unable to conduct a nutritional analysis of advertised foods (due to the quality of the data), the high prevalence of fast food advertisements indicates the nutritional quality of food advertising is likely poor. Previous research has also shown that the foods advertised on television in Canada are high in fat, sugar and salt [30,48].

Future research should investigate the advertising of food and beverage companies, which accounted for a large share of food advertisements, and should benchmark the volume and rates of advertising by company. Research combining data licensed from Numerator (a company that licenses data on actual exposure to television programming and advertising) and CRTC program log data is necessary to examine whether the targeted audience reported by broadcasters are consistent with the children and adolescents’ actual exposure to these programs and advertisements. The CRTC program log data should also be systematically examined for consistency of target age classification of programs, across and within television stations. Furthermore, future studies will need to consider the large fluctuations in rates of food and beverage advertising we observed across the year.

## 5. Conclusions

In Canada, food and beverage products and services remain heavily advertised during television programs targeted at young people, despite the food and advertising industry’s voluntary, self-regulatory codes and initiatives. The current consumer complaint-based process used by Ad Standards Canada, to restrict non-compliant food advertisements, also fails to identify the overt violations by numerous television broadcasters in Canada. More stringent and restrictive legislation, such as Bill S−228 – The Child Health Protection Act, is needed to protect young people from the far-reaching health effects of food and beverage advertising. Federal intervention will enable children across Canada to reap the benefits of food advertising restrictions currently experienced by children in Québec.

## Figures and Tables

**Table 1:** Annual frequency, average rate, and duration of food advertising by target age groups, across all Canadian television stations, in 2018.

	Frequency of Food Advertisements n (%)	Television Stations (n)	Total Food Advertising Duration (hours)	Total Programming Duration (hours)	Food Advertising Rate (n/hour) ^1^		β ^2^	*p*-Value
					Mean	Min-Max		
Preschoolers	10,123 (0.2)	65	60.0	61,823.6	0.6	0–12.2	−3.5	<0.0001
Children	193,888 (3.1)	71	981.4	70,983.7	1.5	0–77.1	−2.6	<0.0001
Adolescents	65,563 (1.0)	106	332.7	25,814.2	3.3	0–21.5	−0.7	<0.0001
Adults	6,074,983 (95.7)	269	31,954.4	1,561,240.5	4.1	0–19.0	Ref.^3^	-

^1^ Significant differences in annual food advertising rates (across all stations) between target age groups was determined by simple linear regression model. *R^2^* = 0.139, *p* < 0.0001. ^2^ β for regression intercept is 4.08 where *p* < 0.0001. ^3^ Ref. = Reference.

**Table 2:** Average age-specific rates of food advertising (n/hr) across 15 Canadian child-specialty television stations ^1^, in 2018.

Food Advertising Rate (n/hr) Mean (SD)				
Child Specialty Stations	Program Target Age Group			
	Preschoolers	Children	Adolescents	Adults
ABC Spark	-	10.0(2.2)	10.9(1.6)	10.7(1.4)
Cartoon Network	-	3.5(1.2) *	1.9(1.8) *	8.8(2.6)
Disney Channel (English)	2.0(1.1) *	3.2(1.3) *	2.3(0.9) *	5.0(2.8)
Disney Channel (French)	0.0(0.0)	0.6(0.4)	-	0.8(0.8)
Disney Junior	0.0(0.0)	0.0(0.0)	-	0.0(0.0)
Disney XD	-	3.7(1.2)	-	-
Family Channel	1.0(1.3) *	2.4(1.3) *	5.4(2.1) *	5.7(2.3)
Family Jr.	0.0(0.0)	0.0(0.0)	0.0(0.0)	-
Nickelodeon	0.0(0.0)	4.5(2.2)	-	2.0(0.0)
Teletoon (English)	0.0(0.0) *	7.0(2.1) *	9.1(4.9)	8.7(2.4)
Télétoon (French)	-	1.3(0.5) *	3.2(1.8)	5.5(1.2)
Treehouse TV	0.0(0.0)	0.0(0.0) *	-	0.0(0.1)
VRAK	-	3.1(0.9)	3.1(0.8)	2.9(0.8)
YOOPA	0.0(0.0)	0.0(0.0)	-	0.0(0.0)
YTV	3.0(4.2) *	6.5(1.4) *	15.3(4.2) *	12.2(2.0)

^1^ Hyphens indicate no programming was found on the television station for the target age group. *Significant differences between food advertising rates during adult programming and the specified age group programming was found in specific multiple linear regression model at *p* < 0.05 level, using an ESTIMATE statement (Supplementary Tables S2 and S3, respectively).

**Table 3:** Average food advertising rates (n/hr) among top five non-child-specialty Canadian television stations, per target program age group, in 2018.

Food Advertising Rate (n/hr) Per Program Target Age Group							
Preschoolers		Children		Adolescents		Adults	
Station	Mean (SD)	Station	Mean (SD)	Station	Mean (SD)	Station	Mean (SD)
OMNI Toronto	11.0(1.3) *	Citytv Toronto	20.0(0) *	Lifetime	18.8(0) *	The Cooking Channel	15.3(1.8)
Sportsnet One	7.1(0) *	W Network	17.7(24.2)	Citytv Vancouver	16.0(0) *	Zeste	12.1(2.1)
BBC Canada	6.9(2.0)	Showcase	11.5(2.4) *	Slice	12.5(2.9) *	Lifetime	11.8 (1.8)
Global Kelowna	4.1(0) *	Movie Time	10.5(2.7)	Adult Swim	9.2(2.2)	Food Network	11.6(2.3)
Nat Geo Wild	3.4(1.3) *	Yes TV Burlington	7.1(3.2) *	Cosmopolitan TV	9.1(5.3)	Citytv Montreal	11.0(1.3)

* Significant differences between food advertising rates during adult programming and the specified age group programming was found in specific multiple linear regression model at *p* < 0.05 level, using an ESTIMATE statement (Supplementary Tables S2 and S3, respectively).

**Table 4:** Age-specific frequency and proportion of food advertising, by food category, on Canadian Table 2018.

Food Category	Frequency of Food Advertisements n (%) ^1^				Chi Square Results (df)
	Program Target Age Group				
	Preschoolers	Children	Adolescents	Adults	
Food Delivery Services	0 (0)	193 (0.1)	164 (0.3)	26075 (0.4)	X^2^(21) *p* <0.0001
Diet Products & Services	19 (0.2)	15 (0.01)	90 (0.1)	37894 (0.6)	
Fast Food	1267 (12.5)	39673 (20.5)	33475 (51.1)	2370025 (39.0)	
Food & Beverage Products	5432 (53.7)	142451 (73.5)	27268 (41.6)	2886628 (47.5)	
Retail Outlets	2390 (23.6)	6315 (3.3)	2024 (3.1)	376570 (6.2)	
Restaurants	505 (5.0)	2676 (1.4)	1853 (2.8)	307821 (5.1)	
Meal Kits & Subscriptions	510 (5.0)	2565 (1.3)	689 (1.1)	68845 (1.1)	
Unknown	0 (0)	0 (0)	0 (0)	1125 (0.02)	
Total	10123 (100)	193888 (100)	65563 (100)	6074983 (100)	

^1^ Proportion of food advertisements by food category calculated within age-groups.

## Supplementary Materials

The following are available online at https://www.mdpi.com/1660-4601/17/6/1999/s1, Table S1: Average age-specific food advertising (n/hr) across 271 Canadian television stations in 2018, Table S2: Specific multiple linear regression model testing the effect of target program age, television station and month on rate of food advertising across 31 Canadian television stations in 2018, and Table S3: Station-specific food advertising rate comparisons between target age groups.

## References

[1] Childhood Obesity. https://www.canada.ca/en/public-health/services/childhood-obesity/childhood-obesity.html.

[2] Overweight and Obesity in Children and Adolescents: Results from the 2009 to 2011 Canadian Health Measures Survey. https://www150.statcan.gc.ca/n1/pub/82-003-x/2012003/article/11706-eng.htm.

[3] Afshin A., Forouzanfar M.H., Reitsma M.B., Sur P., Estep K., Lee A., Marczak L., Mokdad A.H., Moradi-Lakeh M., GBD 2015 Obesity Collaborators (2017). Health Effects of Overweight and Obesity in 195 Countries over 25 Years. N. Engl. J. Med..

[4] Ebbeling C., Pawlak D., Ludwig D. (2002). Childhood obesity: Public-health crisis, common sense cure. Lancet.

[5] Pabayo R., Spence J., Casey L., Storey K. (2012). Food Consumption Patterns in Preschool Children. Can. J. Diet. Pract. Res..

[6] Prowse R. (2017). Food marketing to children in Canada: A settings-based scoping review on exposure, power and impact. HPCDP.

[7] Set of Recommendations on the Marketing of Foods and Non-alcoholic Beverages to Children. https://apps.who.int/iris/bitstream/handle/10665/44416/9789241500210_eng.pdf;jsessionid=A3F700E3E9490DB3B2C3543450A2632E?sequence=1.

[8] Kraak V.I., Vandevijvere S., Sacks G., Brinsden H., Hawkes C., Barquera S., Lobstein T., Swinburn B.A. (2016). Progress achieved in restricting the marketing of high-fat, sugary and salty food and beverage products to children. Bull. World Health Organ..

[9] Communications Monitoring Report 2018. https://crtc.gc.ca/pubs/cmr2019-en.pdf.

[10] Cairns G., Angus K., Hastings G., Caraher M. (2013). Systematic Reviews of the Evidence on the Nature, Extent and Effects of Food Marketing to Children. A Retrospective Summary. Appetite.

[11] Institute of Medicine Committee on Food Marketing and the Diets of Children and Youth (2006). Food Marketing to Children and Youth: Threat or Opportunity?.

[12] Kelly B., Vandevijvere S., Ng S., Adams J., Allemandi L., Bahena-Espina L., Barquera S., Boyland E., Calleja P., Carmona-Garcés I.C. (2019). Global benchmarking of children’s exposure to television advertising of unhealthy foods and beverages across 22 countries. Obes. Rev..

[13] Potvin Kent M., Martin C.L., Kent E.A. (2014). Changes in the volume, power and nutritional quality of foods marketed to children on television in Canada. Obesity.

[14] Elliott C., Cook B. (2013). Not so great: Ten important myths about food advertising targeted to children in Canada. Child. Obes..

[15] Potvin Kent M., Wanless A. (2014). The influence of the Children’s Food and Beverage Advertising Initiative: Change in children’s exposure to food advertising on television in Canada between 2006–2009. Int. J. Obes..

[16] Popkin B.M., Doak C.M. (1998). The obesity epidemic is a worldwide phenomenon. Nutr. Rev..

[17] Story M., French S. (2014). Food Advertising and Marketing Directed at Children and Adolescents in the US. Int. J. Behav. Nutr. Phys. Act..

[18] Story M., Neumark-Sztainer D., French S. (2002). Individual and environmental influences on adolescent eating behaviors. J. Acad. Nutr. Diet..

[19] Fleck F. (2004). WHO resists food industry pressure on its diet plan. BMJ.

[20] Sadeghirad B., Duhaney T., Motaghipisheh S., Campbell NR C., Johnston B.C. (2016). Influence of unhealthy food and beverage marketing on children’s dietary intake and preference: A systematic review and meta-analysis of randomized trials. Obes. Rev..

[21] Dalton M.A., Longacre M.R., Drake K.M., Cleveland L.P., Harris J.L., Hendricks K., Titus L.J. (2017). Child-targeted fast-food television advertising exposure is linked with fast-food intake among pre-school children. Public Health Nutr..

[22] Chacon V., Letona P., Barnoya J. (2013). Child-oriented marketing techniques in snack food packages in Guatemala. BMC Public Health.

[23] Freeman B., Kelly B., Vandevijvere S., Baur L. (2016). Young adults: Beloved by food and drink marketers and forgotten by public health?. Health Promot. Int..

[24] Fleck F. (2003). WHO challenges food industry in report on diet and health. BMJ.

[25] Reducing the Impact of Marketing of Foods and Non-Alcoholic Beverages on Children. https://www.who.int/elena/titles/guidance_summaries/food_marketing_children/en/.

[26] Canadian Children’s Food and Beverage Advertising Initiative. https://adstandards.ca/wp-content/uploads/2018/11/CCFBAI_EN-Nov-2018.pdf.

[27] The Children’s Broadcast Advertising Clearance Guide. https://adstandards.ca/wp-content/uploads/2018/05/kidsGuide.pdf.

[28] Broadcast Code for Advertising to Children. https://adstandards.ca/wp-content/uploads/2018/09/broadcastCodeForAdvertisingToChildren.pdf.

[29] TV and Radio Advertising Basics. https://crtc.gc.ca/eng/television/publicit/publicit.htm.

[30] Mulligan C., Labonté M.È., Vergeer L., L’Abbé M. (2018). Assessment of the Canadian Children’s Food and Beverage Advertising Initiative’s Uniform Nutrition Criteria for Restricting Children’s Food and Beverage Marketing in Canada. Nutrients.

[31] The Canadian Children’s Food and Beverage Advertising Initiative: 2017 Compliance Report. https://adstandards.ca/wp-content/uploads/2018/11/Ad-Standards-CAI-Report-2017-EN.pdf.

[32] Uniform Nutrition Criteria. https://adstandards.ca/about/childrens-advertising-initiative/uniform-nutrition-criteria/.

[33] CAI Participant Commitments. https://adstandards.ca/about/childrens-advertising-initiative/participant-commitments/.

[34] Potvin Kent M., Dubois L., Wanless A. (2012). A nutritional comparison of foods and beverages marketed to children in two advertising policy environments. Obesity.

[35] Potvin Kent M., Smith J.R., Pauzé E., L’Abbé M. (2018). The effectiveness of the food and beverage industry’s self-established uniform nutrition criteria at improving the healthfulness of food advertising viewed by Canadian children on television. Int. J. Behav. Nutr. Phys. Act..

[36] Potvin Kent M., Dubois L., Wanless A. (2011). Self-regulation by industry of food marketing is having little impact during children’s preferred television. Pediatr. Obes..

[37] Bill S-228: An. Act. to Amend the Food and Drugs Act. http://www.parl.ca/DocumentViewer/en/42-1/bill/S-228/third-reading.

[38] Government of Canada Television Program. Logs..

[39] Television Broadcasting Regulations, 1987. https://laws-lois.justice.gc.ca/eng/regulations/SOR-87-49/.

[40] Broadcasting Regulatory Policy CRTC 2017-279-1. https://crtc.gc.ca/eng/archive/2017/2017-279-1.pdf.

[41] Broadcasting Regulatory Policy CRTC 2016-224. https://crtc.gc.ca/eng/archive/2016/2016-224.pdf.

[42] Giveaway. https://en.oxforddictionaries.com/definition/giveaway.

[43] Merchandising. https://en.oxforddictionaries.com/definition/merchandising.

[44] Decision CRTC 94-655. https://crtc.gc.ca/eng/archive/1994/db94-655.htm.

[45] Rules for Unsolicited Telecommunications Made on Behalf of Political Entities. https://crtc.gc.ca/eng/phone/telemarketing/politi.htm.

[46] Broadcasting Regulatory Policy CRTC 2010-622. https://crtc.gc.ca/eng/archive/2010/2010-622.pdf.

[47] Broadcasting Regulatory Policy CRTC 2016-146. https://crtc.gc.ca/eng/archive/2016/2016-146.htm.

[48] (2013). SAS/ACCESS® 9.4 Interface to ADABAS (for Windows).

[49] Television Program. Logs Reference Tables. https://applications.crtc.gc.ca/OpenData/Television%20Logs/ReferenceTables/ReferenceTables.zip.

[50] Television Program. Logs Call Signs. https://applications.crtc.gc.ca/OpenData/Television%20Logs/STAR2/Call%20Signs.xlsx.

[51] Kelly B., Halford J.C., Boyland E.J., Chapman K., Bautista-Castaño I., Bautista-Castaño I., Berg C., Caroli M., Cook B., Coutinho J.G. (2010). Television food advertising to children: A global perspective. Am. J. Public Health..

[52] Media and Technology Habits of Canadian Youth. https://rocketfund.ca/wp-content/uploads/2017/01/Youth-Media-Tech-ShawRocketFund-Sept19-2014.pdf.

[53] Truman E., Elliott C. (2019). Identifying food marketing to teenagers: A scoping review. Int. J. Behav. Nutr. Phys. Act..

[54] Mcphail D., Chapman G.E., Beagan B.L. (2011). Too Much of That Stuff Can’t Be Good: Canadian Teens, Morality, and Fast Food Consumption. Soc. Sci. Med..

[55] Federal Trade Commission A Review of Food Marketing to Children and Adolescents. https://www.ftc.gov/sites/default/files/documents/reports/review-food-marketing-children-and-adolescents-follow-report/121221foodmarketingreport.pdf.

[56] Czoli C., Pauzé E., Potvin Kent M. (2020). Exposure to food and beverage advertising on television among Canadian adolescents, 2011 to 2016. Nutrients.

[57] Russell S.J., Croker H., Viner R.M. (2019). The effect of screen advertising on children’s dietary intake: A systematic review and meta-analysis. Obes. Rev..

[58] Andreyeva T., Kelly I., Harris J. (2011). Exposure to food advertising on television: Associations with children’s fast food and soft drink consumption and obesity. Econ. Hum. Biol..

[59] Majabadi H.A., Solhi M., Montazeri A., Shojaeizadeh D., Nejat S., Farahani F.K., Djazayeri A. (2016). Factors Influencing Fast-Food Consumption Among Adolescents in Tehran: A Qualitative Study. Iran. Red Crescent Med. J..

[60] Emond J.A., Longacre M.R., Drake K.M., Titus L.J., Hendricks K., MacKenzie T., Harris J.L., Carroll J.E., Cleveland L.P., Gaynor K. (2019). Influence of child-targeted fast food TV advertising exposure on fast food intake: A longitudinal study of preschool-age children. Appetite.

[61] Robinson T.N., Borzekowski D.L., Matheson D.M., Kraemer H.C. (2007). Effects of Fast Food Branding on Young Children’s Taste Preferences. JAMA Pediatr..

[62] Elliott C., Den Hoed D., Conlon R. (2013). Food Branding and Young Children’s Taste Preferences: A Reassessment. Can. J. Public Health.

[63] Boyland E.J., Kavanagh-Safran M., Halford J.C.G. (2015). Exposure to ‘healthy’ fast food meal bundles in television advertisements promotes liking for fast food but not healthier choices in children. Br. J. Nutr..

[64] Bahadoran Z., Mirmiran P., Azizi F. (2016). Fast Food Pattern and Cardiometabolic Disorders: A Review of Current Studies. Health Promot. Perspect..

