# Supplementary material for: Food and Beverage Advertising to Children and Adolescents on Television: A Baseline Study
Source: Int J Environ Res Public Health. 2020 Mar 18;17(6):1999. doi: 10.3390/ijerph17061999 (PMC7142724; doi:10.3390/ijerph17061999)
Supplement: Supplementary file 1 [file ijerph-17-01999-s001.pdf]

Supplementary Table S1: Average age-specific food advertising (n/hr) across 271 Canadian Table 2018.

Television Station (n = 271)	Food advertising rate (n/hr) per target age group							
	Preschoolers		Children		Adolescents		Adults	
	Mean	SD	Mean	SD	Mean	SD	Mean	SD
A.Side TV	-	-	0	0	-	-	0.35	0.49
ABC Spark	-	-	9.96	2.15	10.86	1.58	10.73	1.35
AMI-TV	-	-	-	-	-	-	0	0
ATN Aastha TV	0	-	-	-	-	-	0.48	0.26
ATN DD Sports	-	-	-	-	-	-	0	0
ATN Punjabi 5	-	-	-	-	-	-	0.4	0.06
ATN South Asian Television	-	-	-	-	-	-	0.57	0.29
Aboriginal Peoples Television	0.02	0.03	0.2	0.3	0.44	0.48	0.65	0.67
Adult Swim	-	-	-	-	9.16	2.2	10.1	2.35
All TV	-	-	0.06	0.09	-	-	0.37	0.07
Animal Planet	-	-	-	-	-	-	3.32	1.4
Asian Television Network ATN	-	-	-	-	-	-	0.17	0.1
BBC Canada	6.85	1.99	-	-	6.97	3.7	6.91	2.06
BBC Earth	-	-	-	-	-	-	0.98	0.74
BC News 1	-	-	-	-	-	-	1.81	0.55
BNN	-	-	-	-	-	-	0.16	0.24
Book Television	-	-	-	-	-	-	2.41	1.5
Bravo!	-	-	-	-	-	-	2.57	0.38
CASA	-	-	-	-	-	-	5.39	0.94
CBC Calgary	0	0	0	0	-	-	5.31	1.03
CBC Charlottetown	0	0	0	0	-	-	4.84	1.49
CBC Edmonton	0	0	0	0	-	-	5.31	1.11
CBC Fredericton	0	0	0	0	-	-	5.26	1.36
CBC Halifax	0	0	0	0	-	-	5.01	1.42
CBC Montreal	0	0	0	0	-	-	4.69	1.08
CBC News	0	0	0	0	-	-	3.9	1.04
CBC News Networks	-	-	-	-	-	-	1.55	0.32
CBC Ottawa	0	0	0	0	-	-	5.2	0.96
CBC Regina	0	0	0	0	-	-	5.27	0.95
CBC St. John's	0	0	0	0	-	-	4.94	1.03
CBC Toronto	0	0	0	0	-	-	4.87	1.09
CBC Vancouver	0	0	0	0	-	-	5.23	0.91
CBC Windsor	0	0	0	0	-	-	4.75	1.04
CBC Winnipeg	0	0	0	0	-	-	5.07	0.91
CBC Yellowknife	0	0	0	0	-	-	3.01	0.71
CHEK Media	-	-	0	0	-	-	1.69	0.73
CMT	-	-	-	-	-	-	10.14	1.54
Yes TV Burlington	-	-	7.05	3.2	-	-	2.25	0.83
Yes TV Calgary	-	-	3.23	0.8	-	-	4.06	0.63
Yes TV Edmonton	-	-	2.09	0.77	-	-	3.82	0.83
CTV Ottawa	-	-	-	-	1.33	1.26	3.52	0.77
CTV Winnipeg	-	-	-	-	1.33	1.26	2.94	0.73
CTV 2 Alberta	-	-	-	-	0.99	0.92	2.9	0.93
CTV 2 Atlantic	-	-	-	-	1.53	1.43	3.38	0.48
CTV Atlantic (Moncton, NB)	-	-	-	-	1.33	1.26	3.31	0.8
CTV Atlantic (Saint John, NB)	-	-	-	-	1.33	1.26	3.31	0.8
CTV Atlantic (Halifax, NS)	-	-	-	-	1.33	1.26	3.11	0.81
CTV Atlantic (Sydney, NS)	-	-	-	-	1.33	1.26	3.08	0.8
CTV Calgary	-	-	-	-	1.33	1.26	4.34	1.23
CTV Edmonton	-	-	-	-	1.33	1.26	4.51	1.22

CTV Kitchener	-	-	-	-	1.33	1.26	3.62	0.76
CTV Lethbridge	-	-	-	-	1.33	1.26	4.33	1.22
CTV Lloydminster	-	-	-	-	1.11	0.8	1.01	0.36
CTV Montreal	-	-	-	-	1.33	1.26	2.8	0.81
CTV News Channel (Toronto, ON)	-	-	-	-	-	-	0.74	0.46
CTV Northern Ontario	-	-	-	-	2	0.71	3.68	0.65
CTV Prince Albert	-	-	-	-	1.33	1.26	3.09	0.77
CTV Red Deer	-	-	-	-	1.33	1.26	4.59	1.21
CTV Regina	-	-	-	-	1.33	1.26	2.99	0.69
CTV Saskatoon	-	-	-	-	1.33	1.26	3.17	0.75
CTV Sault St Marie	-	-	-	-	2	0.71	3.51	0.68
CTV Sudbury	-	-	-	-	1.33	1.26	3.4	0.72
CTV Thunder Bay	-	-	-	-	0	0	6.02	1.48
CTV Timmins	-	-	-	-	2	0.71	3.68	0.66
CTV Toronto	-	-	-	-	1.33	1.26	3.42	0.76
CTV Two	-	-	-	-	2.88	1.2	1.11	0.24
CTV Two Barrie	-	-	-	-	0	-	2.77	0.62
CTV Two Dawson Creek	-	-	0	-	0.04	0.13	1.75	0.56
CTV Two London	-	-	-	-	0	0	2.66	0.59
CTV Two Pembroke	-	-	-	-	0.53	0.56	2.79	0.5
CTV Two Vancouver	-	-	-	-	2.5	3.12	4.67	0.96
CTV Two Vancouver Island	-	-	-	-	0.92	0.77	3.18	0.63
CTV Two Windsor	-	-	-	-	0	0	2.65	0.56
CTV Yorkton	-	-	-	-	1.33	1.26	2.97	0.72
CablePulse 24 Toronto	-	-	-	-	-	-	0.84	0.28
Canal D	-	-	-	-	-	-	2.95	0.79
Canal Vie	-	-	-	-	-	-	2.76	0.66
Cartoon Network	-	-	3.48	1.23	1.91	1.79	8.83	2.63
Channel Zero Hamilton	-	-	-	-	2.9	2.46	2.6	0.7
Citytv	-	-	-	-	-	-	0.87	0.29
Citytv Montreal	-	-	-	-	-	-	11	1.29
Citytv Toronto	-	-	20	-	-	-	8.5	1.15
Citytv Calgary	-	-	-	-	-	-	9.94	1.21
Citytv Edmonton	-	-	-	-	-	-	8.19	0.91
Citytv Nothern BC	-	-	1.33	0.94	2.22	1.67	1.02	0.49
Citytv Vancouver	-	-	-	-	16	-	10.04	1.67
Citytv Winnipeg	-	-	-	-	-	-	10.14	1.46
Comedy	-	-	-	-	-	-	3.76	0.77
Comedy Gold	-	-	-	-	-	-	3.41	1.57
Community Focus TV	-	-	-	-	-	-	0	0
Cosmopolitan TV	-	-	-	-	9.06	5.28	7.56	1.94
Cottage Life	-	-	-	-	-	-	3.94	0.78
Crime + Investigation	-	-	-	-	-	-	7.35	1.22
DTOUR	-	-	-	-	-	-	9.54	1.23
Daystar Canada	-	-	0	0	0	0	0	0
DejaView	-	-	-	-	5.64	1.72	5.77	1.59
Discovery	-	-	-	-	-	-	2.39	0.39
Discovery Science	-	-	-	-	-	-	2.1	1.06
Discovery Velocity	-	-	-	-	-	-	2.69	1.33
Disney Channel English	1.95	1.1	3.18	1.3	2.34	0.91	4.95	2.76
Disney Channel French	0	0	0.57	0.43	-	-	0.77	0.75
Disney Junior	0	0	0	0	-	-	0	0
Disney XD	-	-	3.7	1.22	-	-	-	-
Diy Network	-	-	-	-	-	-	6.42	1.51
Documentary Channel	-	-	-	-	-	-	0.07	0.14
E!	-	-	-	-	-	-	3.74	0.68
ESPN Classic	-	-	-	-	-	-	2.44	1.39
FPTV (Festival Portuguese Tele	-	-	-	-	-	-	0.59	0.14

FX	-	-	-	-	-	-	7.74	1.14
FXX	-	-	-	-	-	-	8.57	0.91
Fairchild TV	-	-	-	-	-	-	0.47	0.2
Fairchild TV 2 HD	-	-	-	-	-	-	1.27	0.32
FaithTV	-	-	-	-	-	-	0	0
Family Channel	0.99	1.3	2.44	1.28	5.4	2.05	5.65	2.32
Family Jr.	0	0	0	0	0	-	-	-
Fashion Television Channel	-	-	-	-	3.39	1.59	3.35	1.36
Fight Network	-	-	-	-	1.07	0.27	0.66	0.3
Food Network	-	-	-	-	7	-	11.63	2.27
Game TV	-	-	-	-	5.21	2.16	3.88	1.3
Games+	-	-	-	-	0.38	0.33	0.45	0.36
Global Edmonton	-	-	-	-	-	-	9.31	1.15
Global Calgary	-	-	-	-	-	-	9.03	0.95
Global Durham/Oshawa	0	0	-	-	0	0	0	0.01
Global Halifax	-	-	-	-	-	-	10.79	1.34
Global Kelowna	4.13	-	-	-	-	-	7.92	2.39
Global Kingston	0.84	1.45	-	-	-	-	1.14	0.33
Global Lethbridge	-	-	-	-	-	-	10.34	1.06
Global Lloydminster	-	-	-	-	1.53	-	1.36	0.49
Global Montreal	-	-	-	-	-	-	10.24	1.35
Global Peterborough	0.81	0.93	-	-	-	-	1.25	0.31
Global Prescott	1.11	1.15	-	-	-	-	1.5	0.62
Global Regina	-	-	-	-	-	-	10.86	1.35
Global Saskatoon	-	-	-	-	-	-	10.29	1.33
Global Thunder Bay	-	-	-	-	-	-	5.86	1.12
Global Toronto	-	-	-	-	-	-	8.52	0.83
Global Vancouver	-	-	-	-	-	-	8	0.97
Global Winnipeg	-	-	-	-	-	-	9.39	1.48
Gusto TV	-	-	-	-	-	-	3.68	0.77
H2	-	-	-	-	5.45	2.45	5.96	1.76
HGTV	-	-	3.91	0.56	-	-	5.95	1.04
HIFI	-	-	0.2	0.19	-	-	1.36	0.83
Historia	-	-	-	-	-	-	7.48	0.88
History Television	-	-	-	-	-	-	6.11	1.29
ICI Explora	1.97	0.85	-	-	-	-	1.47	0.54
ICI RDI	-	-	-	-	-	-	4.32	1.21
Ici Montreal	-	-	-	-	-	-	2.41	0.98
Ici Radio-Canada Télé	0.36	0.24	0.28	0.25	3.39	1.76	3.94	0.5
Investigation	-	-	-	-	-	-	3.49	1.25
Investigation Discovery	-	-	-	-	-	-	3.78	1.05
Le Canal Nouvelle	-	-	-	-	-	-	3.42	0.69
Leafs TV	-	-	-	-	-	-	0.07	0.08
Lifetime	-	-	-	-	18.79	-	11.76	1.84
Love Nature	-	-	0	0	-	-	0	0
MOI&cie	-	-	-	-	-	-	6.53	2.06
MTV Canada	-	-	-	-	4.67	1	4.31	0.62
MTV2	-	-	-	-	5.19	2.16	5.13	2.19
Makeful TV	-	-	4.18	1.49	-	-	5.07	1.59
Max HD	-	-	-	-	-	-	3.44	0.46
Mediaset Italia	-	-	-	-	-	-	0.62	0.11
Miracle Channel	-	-	0	0	0	0	0	0
Movie Time	-	-	10.45	2.72	8.53	2.15	9	1.06
MuchMusic	-	-	-	-	2.69	3.27	4.47	0.91
MusiquePlus MP	-	-	-	-	-	-	3.47	0.76
NBA TV	-	-	-	-	-	-	0.42	0.34
NTV(Newfoundland Television) S	-	-	-	-	-	-	10.5	1.48
Nat Geo Wild	3.35	1.34	-	-	4.61	2.07	4.84	1.52

National Geographic Channel	-	-	-	-	7.55	2.32	8.03	1.79
New Tang Dynasty (NTD) Televis	-	-	-	-	-	-	0	0
Nickelodeon	0	0	4.53	2.18	-	-	2	-
OMNI Regional	-	-	-	-	-	-	7.85	1.48
Odyssey	-	-	-	-	-	-	0	0
Omni Calgary	-	-	-	-	-	-	7.85	1.48
Omni Edmonton	-	-	-	-	-	-	7.93	1.42
Omni Toronto	11.02	1.25	-	-	-	-	5.51	0.7
Omni Vancouver	-	-	-	-	-	-	6.54	1.43
One: the Body, Mind & Spirit	-	-	-	-	-	-	1.7	1.11
OutTV Vancouver	-	-	-	-	0	0	0	0
Outdoor Life Network	-	-	-	-	-	-	9.07	1.43
Prise 2	-	-	-	-	6.67	1.61	4.54	1.14
RDS Info	-	-	-	-	-	-	1.13	0.22
RDS, Réseau des sports	-	-	-	-	-	-	1.85	0.33
Rewind	-	-	-	-	-	-	0.11	0.14
SRC Rimouski, Quebec	0.73	0.4	0.39	0.35	3.43	1.77	6.16	1
SRC Edmonton	0.44	0.34	0.29	0.25	4.28	2.27	5.28	1.21
SRC Moncton	0.38	0.21	0.25	0.22	6.3	4.48	5.64	0.95
SRC Montreal	0.4	0.23	0.3	0.27	4.63	2.22	4.77	0.47
SRC Ottawa	0.42	0.2	0.33	0.21	4.05	2.19	5.23	0.63
SRC Quebec City	0.46	0.28	0.32	0.28	4.81	1.94	6.19	0.87
SRC Regina	0.44	0.34	0.29	0.25	4.28	2.27	5.3	1.19
SRC Rivière-du-Loup	-	-	-	-	-	-	0.13	0.18
SRC Saguenay	0.56	0.29	0.39	0.41	4	1.64	6.07	1.01
SRC Sherbrooke	0.42	0.3	0.29	0.25	4.47	2.55	5.75	0.79
SRC Toronto	0.44	0.34	0.3	0.26	3.65	1.77	5.51	1.24
SRC Trois-Rivières	0.39	0.26	0.3	0.27	3.77	1.92	5.39	0.77
SRC Vancouver	0.44	0.34	0.29	0.26	4.55	2.54	5.48	1.27
SRC Winnipeg	0.44	0.34	0.29	0.25	4.6	2.49	5.43	1.23
Saskatchewan Communications Ne	0	0	0	0	0	0	8.63	0.81
Showcase	-	-	11.5	2.41	5.6	-	8.85	1.39
Silver Screen Classics	-	-	-	-	-	-	0.06	0.09
Slice	-	-	-	-	12.51	2.92	9.47	2.43
Smithsonian Channel	-	-	-	-	-	-	1.05	0.44
Space	-	-	-	-	-	-	3.12	0.6
Sportsman Canada	-	-	0	0	0	0	0	0
Sportsnet 360	-	-	-	-	-	-	5.5	1.16
Sportsnet East	-	-	-	-	-	-	5.07	0.83
Sportsnet One	7.13	-	-	-	-	-	3.55	1.16
Sportsnet Ontario	-	-	-	-	-	-	4.56	1.68
Sportsnet Pacific	-	-	-	-	-	-	3.54	2.71
Sportsnet West	-	-	-	-	-	-	5.58	0.98
Super Channel	-	-	-	-	-	-	0	0
Séries+	-	-	-	-	-	-	7.62	0.94
TFO	0	0	0.08	0.13	0.05	0.14	0.01	0.02
TNS 1	-	-	-	-	-	-	3.09	0.43
TRC, the Rural Channel	-	-	-	-	-	-	0	0
TSN - The Sports Network	-	-	-	-	-	-	3.54	0.47
TSN 2	-	-	-	-	-	-	2.36	0.64
TSN 3	-	-	-	-	-	-	3.14	0.54
TSN 5	-	-	-	-	-	-	3.62	0.54
TV5 Québec	-	-	-	-	2.74	1.02	1.64	0.43
TVA Carleton	-	-	-	-	-	-	0.1	0.14
TVA Gatineau	-	-	-	-	4.51	3.09	1.14	0.18
TVA Montreal	2.52	0.74	-	-	3.55	0.62	3.59	0.21
TVA Quebec City	3.03	1.45	-	-	3.88	0.62	3.68	0.32
TVA Rimouski	2.18	0.26	-	-	3.06	0.38	3.15	0.33

TVA Rivière-du-Loup	-	-	-	-	-	-	0.14	0.22
TVA Saguenay	2.18	0.26	-	-	3.06	0.87	3.18	0.27
TVA Sherbrooke	2.18	0.26	-	-	3.31	0.38	3.53	0.33
TVA Sports	-	-	-	-	-	-	1.88	0.37
TVA Trois Rivières	3.18	1.16	-	-	3.55	0.14	3.27	0.34
TVA Rouyn-Noranda	-	-	-	-	4.47	3.02	0.63	0.21
TVOntario	0	0	0	0	-	-	0	0
Talent Vision	-	-	-	-	-	-	0.35	0.27
Telelatino	-	-	-	-	-	-	5.01	1.09
Teletoon English	0	0	6.97	2.06	9.05	4.93	8.67	2.4
Teletoon French	-	-	1.28	0.51	3.24	1.76	5.47	1.22
The Beautiful Little Channel	-	-	-	-	-	-	0	0
The Cooking Channel	-	-	-	-	-	-	15.34	1.84
The Independent Film Channel C	3.25	3.18	-	-	-	-	3.35	2.06
The Oprah Winfrey Network	-	-	-	-	-	-	6.95	1.1
The Weather Network	-	-	-	-	-	-	2.79	0.42
Treehouse TV	0.02	0.04	0	0.01	-	-	0.02	0.07
Télé-Québec Montreal	1.89	0.64	2.38	0.8	6.05	1.45	5.58	1.07
TéléMag	-	-	-	-	0	0	0.46	0.38
UnisTv Montreal	0	0	0.62	0.28	1.31	0.49	1.33	0.49
Univision Canada	0	0	0	0	4.18	0.81	3.36	0.46
Unknown	-	-	-	-	-	-	3.76	0.38
V (Network)	-	-	-	-	-	-	2.68	0.23
V Gatineau	-	-	-	-	2.17	2	1.51	0.38
V Interactios Inc. Quebec City	-	-	-	-	-	-	3.75	0.62
V Montreal	-	-	-	-	-	-	1.21	0.54
V Rivière-du-Loup	-	-	-	-	-	-	0.1	0.15
V Saguenay	-	-	-	-	-	-	2.94	0.36
V Sherbrooke	-	-	-	-	-	-	3.22	0.39
V Trois-Rivières	-	-	-	-	-	-	2.95	0.36
V Val-d'Or	-	-	0	-	1.86	1.05	0.78	0.46
VRAK	-	-	3.06	0.94	3.1	0.81	2.92	0.8
W Network	-	-	17.71	24.2	4.73	0.64	8	1.55
Wild Tv	-	-	-	-	-	-	0.14	0.33
YOOPA	0	0	0	0	-	-	0	0
YTV	2.98	4.22	6.54	1.38	15.33	4.17	12.17	2.03
Z	-	-	-	-	-	-	3.01	0.62
Zee Cinema Canada	-	-	-	-	-	-	0.4	0.64
Zee Premier Canada	-	-	-	-	-	-	0	0
Zeste	-	-	-	-	-	-	12.11	2.13
Zing	-	-	-	-	-	-	0.09	0.22
ZoomerMedia Limited	-	-	-	-	-	-	0.82	0.43
addikTV	-	-	-	-	-	-	5.36	0.71
fyi	-	-	-	-	4.95	1.57	5.85	1.56
ici ARTV	1.5	0.8	-	-	3.88	1.21	3.08	1.14
travel + escape	-	-	-	-	-	-	4.81	2.07
Évasion	-	-	-	-	-	-	9.65	3.09

Hyphens indicate no programming (i.e. no food advertising rate) was found for the specific age group on the television station; SD = Standard Deviation.

Supplementary Table S2: Specific multiple linear regression model [1] testing the effect of target program age by television station and month on the rate of food advertising across 31 Canadian television stations [2], in 2018.

Parameter	β	Standard Error	*p*-value
**Intercept**	8.516	0.555	<0.0001

**Program Target Age Group**			
Preschoolers	-9.837	1.439	<0.0001
Adolescents	-3.272	1.252	0.0092
Children	1.162	0.942	0.2182
Adults	Reference		
**Television Station**			
ABC Spark	2.739	0.664	<0.0001
Adult Swim	2.170	0.680	0.0015
BBC Canada	-1.087	0.664	0.1021
Yes TV Burlington	-5.747	0.664	<0.0001
Cartoon Network	0.838	0.664	0.2077
Citytv Toronto	0.506	0.664	0.4461
Citytv Vancouver	2.047	0.664	0.0021
Cosmopolitan TV	-0.437	0.664	0.5105
Disney Channel English	-3.038	0.698	<0.0001
Disney Channel French	-7.178	0.680	<0.0001
Disney Junior	-8.479	0.745	<0.0001
Family Channel	-2.344	0.664	0.0004
Family Jr.	-9.250	0.780	<0.0001
Food Network	3.640	0.664	<0.0001
Global Kelowna	-0.077	0.664	0.9078
Lifetime	3.767	0.664	<0.0001
Movie Time	1.078	0.680	0.1131
Nat Geo Wild	-3.160	0.664	<0.0001
Nickelodeon	-5.341	1.722	0.002
Omni Toronto	-2.486	0.664	0.0002
Showcase	0.854	0.664	0.1989
Slice	1.475	0.664	0.0268
Sportsnet One	-4.450	0.664	<0.0001
Teletoon English	0.676	0.664	0.3089
Teletoon French	-2.528	0.664	0.0002
The Cooking Channel	7.349	0.664	<0.0001
Treehouse TV	-7.974	0.664	<0.0001
VRAK	-5.563	0.745	<0.0001
YOOPA	-7.483	1.062	<0.0001
YTV	4.172	0.664	<0.0001
W Network	Reference		
**Month**			
January	0.026	0.439	0.9523
February	-0.513	0.435	0.2385
March	-0.155	0.435	0.7216
June	-0.363	0.432	0.401
July	0.721	0.435	0.0977
August	0.241	0.449	0.592
September	-0.584	0.444	0.1892
October	-1.177	0.449	0.009
November	-1.714	0.444	0.0001
December	-2.480	0.441	<0.0001
April	-0.257	0.432	0.5524
May	Reference		
**Program Target Age Group*Television Station**			
Preschoolers*BBC Canada	9.116	1.624	<0.0001
Preschoolers*Disney Channel English	6.146	1.466	<0.0001
Preschoolers*Disney Channel French	8.333	1.457	<0.0001
Preschoolers*Disney Junior	9.634	1.489	<0.0001
Preschoolers*Family Channel	4.447	1.497	0.0031
Preschoolers*Family Jr.	10.405	1.647	<0.0001
Preschoolers*Global Kelowna	5.193	2.187	0.0179

Preschoolers*Nat Geo Wild	7.661	1.450	<0.0001
Preschoolers*Nickelodeon	6.497	2.151	0.0026
Preschoolers*Omni Toronto	14.545	1.623	<0.0001
Preschoolers*Sportsnet One	12.837	2.197	<0.0001
Preschoolers*Teletoon English	0.511	1.466	0.7274
Preschoolers*Treehouse TV	9.146	1.450	<0.0001
Preschoolers*YOOPA	8.638	1.670	<0.0001
Preschoolers*YTV	Reference		
Adolescents*ABC Spark	3.548	1.274	0.0055
Adolescents*Adult Swim	1.834	1.464	0.2109
Adolescents*BBC Canada	3.616	1.308	0.0059
Adolescents*Cartoon Network	-3.505	1.274	0.0061
Adolescents*Citytv Vancouver	10.701	2.045	<0.0001
Adolescents*Cosmopolitan TV	4.810	1.456	0.001
Adolescents*Disney Channel English	0.803	1.292	0.5345
Adolescents*Family Channel	3.170	1.274	0.0131
Adolescents*Family Jr.	6.548	2.119	0.0021
Adolescents*Food Network	0.656	2.079	0.7525
Adolescents*Lifetime	10.667	2.086	<0.0001
Adolescents*Movie Time	3.113	1.384	0.0248
Adolescents*Nat Geo Wild	3.529	1.289	0.0064
Adolescents*Showcase	0.387	2.086	0.853
Adolescents*Slice	6.783	1.372	<0.0001
Adolescents*Teletoon English	3.812	1.333	0.0044
Adolescents*Teletoon French	2.232	1.703	0.1903
Adolescents*VRAK	3.962	1.362	0.0037
Adolescents*YTV	6.050	1.339	<0.0001
Adolescents*W Network	Reference		
Children*ABC Spark	-1.995	1.047	0.0571
Children*Yes TV Burlington	3.542	1.025	0.0006
Children*Cartoon Network	-6.605	1.025	<0.0001
Children*Citytv Toronto	9.752	1.910	<0.0001
Children*Disney Channel English	-3.035	1.047	0.0039
Children*Disney Channel French	-1.506	1.035	0.146
Children*Disney Junior	-0.771	1.079	0.475
Children*Family Channel	-4.464	1.025	<0.0001
Children*Family Jr.	Reference		
Children*Movie Time	0.293	1.113	0.7927
Children*Nickelodeon	0.618	1.890	0.7438
Children*Showcase	1.566	1.132	0.167
Children*Teletoon English	-2.959	1.025	0.004
Children*Teletoon French	-5.443	1.025	<0.0001
Children*Treehouse TV	-1.273	1.025	0.2147
Children*VRAK	-0.890	1.134	0.4328
Children*YOOPA	-1.767	1.318	0.1803
Children*YTV	-6.881	1.025	<0.0001
Children*W Network	Reference		
Adults*ABC Spark	Reference		
Adults*Adult Swim	Reference		
Adults*BBC Canada	Reference		
Adults*Yes TV Burlington	Reference		
Adults*Cartoon Network	Reference		
Adults*Citytv Toronto	Reference		
Adults*Citytv Vancouver	Reference		
Adults*Cosmopolitan TV	Reference		
Adults*Disney Channel English	Reference		
Adults*Disney Channel French	Reference		
Adults*Disney Junior	Reference		

Adults*Family Channel	Reference		
Adults*Food Network	Reference		
Adults*Global Kelowna	Reference		
Adults*Lifetime	Reference		
Adults*Movie Time	Reference		
Adults*Nat Geo Wild	Reference		
Adults*Nickelodeon	Reference		
Adults*Omni Toronto	Reference		
Adults*Showcase	Reference		
Adults*Slice	Reference		
Adults*Sportsnet One	Reference		
Adults*Teletoon English	Reference		
Adults*Teletoon French	Reference		
Adults*The Cooking Channel	Reference		
Adults*Treehouse TV	Reference		
Adults*VRAK	Reference		
Adults*YOOPA	Reference		
Adults*YTV	Reference		
Adults*W Network	Reference		
**Program Target Age Group*Month**			
Preschoolers*January	0.044	0.884	0.9603
Preschoolers*February	0.579	0.869	0.5058
Preschoolers*March	0.098	0.869	0.9099
Preschoolers*June	0.702	0.846	0.4073
Preschoolers*July	-0.558	0.857	0.5149
Preschoolers*August	-0.040	0.855	0.9631
Preschoolers*September	0.994	0.872	0.2548
Preschoolers*October	1.287	0.875	0.1415
Preschoolers*November	1.448	0.865	0.0946
Preschoolers*December	3.005	0.881	0.0007
Preschoolers*April	0.685	0.868	0.4305
Preschoolers*May	Reference		
Adolescents*January	-2.019	0.906	0.0262
Adolescents*February	-2.043	0.854	0.017
Adolescents*March	-0.730	0.859	0.3958
Adolescents*June	0.143	0.896	0.8732
Adolescents*July	1.156	0.881	0.19
Adolescents*August	0.025	0.885	0.9777
Adolescents*September	0.779	0.842	0.3552
Adolescents*October	1.302	0.872	0.1356
Adolescents*November	0.264	0.909	0.7718
Adolescents*December	-0.061	0.843	0.9423
Adolescents*April	-0.608	0.886	0.4928
Adolescents*May	Reference		
Children*January	-0.232	0.727	0.7499
Children*February	-0.140	0.724	0.8464
Children*March	-0.139	0.732	0.8497
Children*June	0.228	0.728	0.7544
Children*July	-0.658	0.724	0.3639
Children*August	-0.096	0.738	0.8961
Children*September	1.068	0.737	0.1481
Children*October	0.460	0.735	0.5317
Children*November	-0.017	0.744	0.9816
Children*December	0.609	0.730	0.4048
Children*April	0.040	0.737	0.9565
Children*May	Reference		
Adults*January	Reference		
Adults*February	Reference		

Adults*March	Reference
Adults*June	Reference
Adults*July	Reference
Adults*August	Reference
Adults*September	Reference
Adults*October	Reference
Adults*November	Reference
Adults*December	Reference
Adults*April	Reference
Adults*May	Reference

[1] $F_{(127, 640)}$=43.5; p<0.0001; R^2=0.896; [2] 31 television stations (14 child-specialty stations + 17 non-child-specialty stations) included in the specific linear regression model. Disney XD, CityTV Montreal and Zeste were excluded. The symbol "*" indicates interactions.

Supplementary Table S3: Station-specific food advertising rate comparisons between target age groups.

Comparison	Rate Difference [1]	95% Confidence Interval	*p*-value
Preschoolers vs. Adults BBC Canada	−0.03	(−1.92, 1.86)	0.972
Preschoolers vs. Adults Disney Channel English	−3.00	(−4.37, −1.63)	<0.0001
Preschoolers vs. Adults Disney Channel French	−0.82	(−2.15, 0.52)	0.230
Preschoolers vs. Adults Disney Junior	0.48	(−0.98, 1.95)	0.517
Preschoolers vs. Adults Family Channel	−4.70	(−6.24, −3.16)	<0.0001
Preschoolers vs. Adults Global Kelowna	−3.96	(−7.40, −0.51)	0.024
Preschoolers vs. Adults Nat Geo Wild	−1.49	(−2.79, −0.18)	0.025
Preschoolers vs. Adults Nickelodeon	−2.65	(−6.03, 0.73)	0.124
Preschoolers vs. Adults Omni Toronto	5.40	(3.51, 7.29)	<0.0001
Preschoolers vs. Adults Sportsnet One	3.69	(0.22, 7.15)	0.037
Preschoolers vs. Adults Teletoon English	−8.64	(−9.98, −7.30)	<0.0001
Preschoolers vs. Adults Treehouse TV	0.00	(−1.31, 1.30)	0.995
Preschoolers vs. Adults YOOPA	−0.51	(−2.60, 1.57)	0.630
Preschoolers vs. Adults YTV	−9.15	(−11.68, −6.62)	<0.0001
Children vs. Adults ABC Spark	−0.74	(−2.11, 0.63)	0.290
Children vs. Adults Yes TV Burlington	4.80	(3.49, 6.10)	<0.0001
Children vs. Adults Cartoon Network	−5.35	(−6.65, −4.05)	<0.0001
Children vs. Adults Citytv Toronto	11.01	(7.60, 14.42)	<0.0001
Children vs. Adults Disney Channel English	−1.78	(−3.15, −0.41)	0.011
Children vs. Adults Disney Channel French	−0.25	(−1.59, 1.08)	0.712
Children vs. Adults Disney Junior	0.48	(−0.98, 1.95)	0.517
Children vs. Adults Family Channel	−3.21	(−4.51, −1.90)	<0.0001
Children vs. Adults MovieTime	1.55	(−0.01, 3.11)	0.052
Children vs. Adults Nickelodeon	1.87	(−1.51, 5.25)	0.277
Children vs. Adults Showcase	2.82	(1.21, 4.44)	0.001
Children vs. Adults Teletoon English	−1.70	(−3.01, −0.40)	0.011
Children vs. Adults Teletoon French	−4.19	(−5.49, −2.88)	<0.0001

Children vs. Adults Treehouse TV	−0.02	(−1.32, 1.29)	0.979
Children vs. Adults VRAK	0.37	(−1.25, 1.98)	0.657
Children vs. Adults YOOPA	−0.51	(−2.60, 1.57)	0.630
Children vs. Adults YTV	−5.63	(−6.93, −4.32)	<0.0001
Children vs. Adults W Network	1.26	(−0.28, 2.79)	0.108
Adolescents vs. Adults ABC Spark	0.13	(−1.18, 1.43)	0.849
Adolescents vs. Adults Adult Swim	−1.59	(−3.51, 0.33)	0.105
Adolescents vs. Adults Cartoon Network	−6.93	(−8.23, −5.62)	<0.0001
Adolescents vs. Adults Citytv Vancouver	7.28	(3.77, 10.78)	<0.0001
Adolescents vs. Adults Cosmopolitan TV	1.39	(−0.51, 3.28)	0.151
Adolescents vs. Adults Disney Channel English	−2.62	(−3.99, −1.25)	0.000
Adolescents vs. Adults Family Channel	−0.25	(−1.56, 1.05)	0.705
Adolescents vs. Adults Lifetime	7.25	(3.78, 10.71)	<0.0001
Adolescents vs. Adults Slice	3.36	(1.73, 4.99)	<0.0001
Adolescents vs. Adults Teletoon English	0.39	(−1.09, 1.87)	0.604
Adolescents vs. Adults Teletoon French	−1.19	(−3.72, 1.35)	0.357
Adolescents vs. Adults VRAK	0.54	(−1.08, 2.16)	0.512
Adolescents vs. Adults YTV	2.63	(1.09, 4.17)	0.001

[1] Differences in food advertising rates derived from ESTIMATE statement used in specific multiple linear regression model (see Supplementary Table S2 for regression model and parameter estimates).
